# Platelet Glycoproteins and Fibrinogen in Recovery from Idiopathic Sudden Hearing Loss

**DOI:** 10.1371/journal.pone.0086898

**Published:** 2014-01-23

**Authors:** Daniel Weiss, Bruno Neuner, Kerstin Gorzelniak, Alexis Bremer, Claudia Rudack, Michael Walter

**Affiliations:** 1 Department of Otorhinolaryngology, Head and Neck Surgery, University of Münster, Münster, Germany; 2 Department of Anaesthesiology and Intensive Care Medicine, Campus Virchow-Klinikum and Campus Charité-Mitte, Charité – University Medicine, Berlin, Germany; 3 Institute of Laboratory Medicine, Unfallkrankenhaus Berlin, Berlin, Germany; 4 Institute of Laboratory Medicine, Clinical Chemistry and Pathobiochemistry, Campus Virchow-Klinikum, Charité – University Medicine & Labor Berlin – Charité Vivantes Services GmbH, Berlin, Germany; King's College London School of Medicine, United Kingdom

## Abstract

**Background:**

The pathomechanism and location of idiopathic sudden sensorineural hearing loss (ISSHL) is unclear. In a previous case-control study, we found elevated fibrinogen concentrations and a higher prevalence of T allele carriers of the glycoprotein (Gp) Ia C807T polymorphism in ISSHL patients.

**Methodology:**

127 patients with ISSHL (mean age 53.3 years, 48.8% females), who underwent a standard therapy with high dose steroids, pentoxifyllin and sterofundine over 8 days were included. We examined the influence of GpIa genotype and fibrinogen (BclI-, A312-, HaeIII-) genotype and fibrinogen plasma levels on hearing recovery after 8 weeks (change from baseline: 0 dB  =  no recovery, >0 to 10 dB = moderate recovery, >10 dB = good recovery). In a subsample of 59 patients with ISSHL, we further studied the association of platelet glycoprotein GpIa, Ib and IIIa densities on hearing recovery as well as the possible effect-modification of platelet glycoproteins on hearing recovery by plasma fibrinogen.

**Results:**

In univariate analysis, neither the GpIa genotype nor fibrinogen genotype (all p>0.1) but lower fibrinogen levels (p = 0.029), less vertigo (p = 0.002) and lower GpIIIa receptor density (p = 0.037, n = 59) were associated with hearing recovery. In multivariate analysis, fibrinogen significantly modified the effect of GPIa receptor density on good hearing recovery (effect-modification on multiplicative scale OR = 0.45 (95% confidence interval (0.21–0.94)), p = 0.03). GPIb receptor density below the mean was associated with a 2-fold increase in good hearing recovery both in patients with fibrinogen levels above (p = 0.04) as well as in patients with fibrinogen levels below the mean (p = 0.06). There was no indication for an effect-modification (p = 0.97).

**Conclusions:**

The findings suggest a vascular/rheological origin of ISSHL with unique features of thrombosis in the inner ear artery that may include complex interrelationships among platelet glycoproteins and plasma fibrinogen.

## Introduction

Sudden sensorineural hearing loss (SSHL) is a dysfunction of the inner ear characterized by sudden onset and rapid progression of hearing impairment within hours or days. It can occur at every age, but mostly affects elderly people and has an estimated incidence of 10-20/100,000/year [1], [2]. The true incidence however is likely much higher when taking into account an ageing population in industrialized countries and the fact that hearing impairment is often misdiagnosed or regarded as an age-related, unavoidable fate [2], [3]. Hearing impairment is mostly unilateral with varying severity, but can potentially lead to total deafness. It is often accompanied by vertigo, tinnitus and/or ear pressure [4]. At the time of onset, the aetiology of SSHL is usually unknown. In a recent systematic analysis of 23 research papers, Chau et al could demonstrate that 71% of SSHL cases were classified as idiopathic SSHL (ISSHL) because no underlying pathology could be found. In 13% of cases, infection was shown to be the culprit, whereas 5% were attributed to otologic aetiology, 4% to trauma, 3% to hematologic causes, 2% to neoplasia and another 2% to other causes [5].

Hearing recovery in ISSHL varies widely from complete, spontaneous recovery to non-recovery and shows an unpredictable course [6]–[8]. Reduced blood flow in the inner ear, viral infections and autoimmune processes are hypothetical aetiologies for ISSHL. These possible pathomechanisms have been deduced from animal studies and sporadic histological findings [9], but an allocation of one of these mechanisms to individual cases is difficult. Even the exact location of the disorder (vascular, neuronal, inner ear, cerebral) is not clear. One treatment option involves administration of high-dose steroids intravenously, orally or via intratympanic routes. However, the effectiveness of steroids (oral or systemic) for the treatment of ISSHL remains unproven [10].

One hypothetical scenario in ISSHL is a sudden reduction of the blood flow in the labyrinthic artery, which is a functional end artery. The blood flow in the labyrinthic artery is regulated mainly by adrenergic receptors on smooth muscle cells. It is also influenced by plasma viscosity and local regulatory mechanisms, but not by central regulatory mechanisms as in larger vessels. Due to the possible ischemia-like cause of ISSHL, it was hypothesized that risk factors, comparable to those associated with ischaemic or thromboembolic diseases, such as heart disease, stroke and thrombosis [11]–[13], may play an underlying role in disease-aetiology. However, these studies were not conclusive and, in part, contradictory [14], [15]. The strongest argument for a vascular or haemorheological origin of ISSHL is the observation that outcome is significantly improved in ISSHL patients who underwent combined LDL and fibrinogen apheresis [16]. Because raised fibrinogen and LDL cholesterol-levels are risk factors for such vascular diseases as ischaemic heart disease, stroke and other thromboembolic diseases [17], [18], this observation supports the idea that the aetiology of ISSHL is vascular.

We have recently shown that fibrinogen concentrations are significantly elevated in ISSHL patients [19]. We did not, however, find differences in LDL or HDL cholesterol levels between patients and controls, nor could we find a higher incidence of atherothrombotic fibrinogen polymorphisms in ISSHL patients. The only genetic factor that could be allocated to ISSHL was found in the gene encoding glycoprotein Ia (GpIa) [19]. The GpIa-receptor on the surface of thrombocytes is composed of integrin α_2_β_1_, which is involved in the early steps of platelet adhesion. Here we extended our previous studies to investigate the influence of fibrinogen and glycoprotein Ia and related glycoproteins Ib and IIIa on hearing recovery. It is well known that platelet glycoproteins and fibrinogen interact on various levels in complex interrelationships [20]. GpIa, GpIb and GpIIIa are involved in adhesion of platelets to the vessel wall, fibrinogen binding and propagation of the activation process, whereas fibrinogen is intimately involved in final clot formation. We therefore wanted to additionally evaluate whether the effect of platelet glycoproteins on hearing recovery was modified by fibrinogen.

## Materials and Methods

### Patients and Controls

Initially, 142 consecutive German patients with a pantonal, sudden, sensorineural hearing loss (ISSHL) were included in the study: 75 male and 67 female patients aged between 14 and 83 years. Patients have previously been described [16] and were recruited from a cross-sectional study that consecutively selected ISSHL patients of a typical outpatient department. Hearing loss, measured by pure-tone audiometry, was unilateral and did not exceed two weeks of duration in all cases. Patients included had idiopathic hearing loss of at least 60dB in at least 3 frequencies between 125 and 8,000 Hz compared to the healthy ear. A non-idiopathic hearing loss was ruled out by a thorough otorhinolaryngological examination including ear microscopy, magnetic resonance imaging and serological blood investigations for herpes simplex, varizella zoster and borrelia burgdorferi infections. All patients had a negative anamnesis for familial deafness, chronic otological history, trauma, previous ear surgery, or prior sudden deafness. We excluded patients with a medical condition rendering the use of corticosteroids unsafe, such as: pregnancy, poorly controlled diabetes, chronic infections, peptic ulcer, uncompensated heart disease, recent surgery, and psychiatric disease. All patients underwent a standard therapy with high dose steroids (250 mg prednisolone reduced by 25 mg per day), 1000 ml sterofundine and 400 mg pentoxifylline per day over 8 days. Hearing recovery was calculated by comparing the pure-tone thresholds before and 8 weeks after the therapy. We registered hearing recovery [dB] in 4 selected frequencies: 250 Hz, 1,000 Hz, 2,000 Hz and 4,000 Hz. By the 8 week follow-up, 15 patients had dropped out permanently, reducing the final study population to 127 participants. The control group consisted of 81 healthy German subjects (mean age 49.9 +/− 12.6 years) matched for age and sex. The controls were taken from the Prospective Cardiovascular Münster Study (PROCAM). As shown in table 1, the mean age of the ISSHL study participants was 53.3 years, with 48.8% females. Around three-quarters of participants suffered from tinnitus and roughly one-third from vertigo. Compared with age- and sex-matched controls, patients had higher fibrinogen plasma levels (patients: 280.1 +/− 78.9 mg/dl vs. controls: 230.9 mg/dl +/− 107.9; p =  0.001), higher smoking prevalence (patients: 54.8% vs. controls: 17.7%, p<0.001) and a higher percentage of T-allele carriers in the GpIa C807T-polymorphism (patients: 77.9% vs. controls 65.0%, p = 0.041). A subgroup of 59 patients was used for additional monitoring of platelet glycoprotein (GpIa, GPIb, and GPIIIa) receptor-densities. Except for age (patients with glycoprotein receptor analysis were older than patients without glycoprotein receptor analysis, p<0.001) there were no differences regarding basic patientś characteristics, laboratory-results, and hearing recovery between groups that underwent receptor-density analysis and those who did not (table 1).

**Table 1:** Comparison of study participants with and without data on glycoprotein receptor density, n = 127.

Variable		All patients; n = 127	Patients stratified for glycoprotein receptor analysis		
			With analysis; n = 59 (46.5%)	Without analysis; n = 68 (53.5%)	p
Basic patients' characteristics	Age in years, mean (sd)	53.3 (17.1)	59.5 (13.6)	48.0 (18.1)	**< 0.001**
	Female gender, n (%)	62 (48.8)	27 (45.8)	35 (51.5)	0.521
	Smoker, n (%)^&^	68 (54.8)	32 (56.1)	36 (53.7)	0.788
	Tinnitus, n (%)^&&^	91 (75.2)	39 (72.2)	52 (77.6)	0.495
	Vertigo, n (%)^&&^	46 (37.7)	18 (32.7)	28 (41.8)	0.304
Laboratory values	GPIa-gene				
	CC (wild type), n (%)	28 (22.1)	9 (15.3)	19 (27.9)	0.085
	TC or TT, n (%)	99 (77.9)	50 (84.7)	49 (72.1)	
	BcLI-gene				
	CC (wild type), n (%)	82 (64.6)	40 (67.8)	42 (61.8)	0.478
	TC or TT, n (%)	45 (35.4)	19 (32.2)	26 (38.2)	
	A312-gene				
	CC, n (%)	77 (60.6)	33 (55.9)	44 (64.7)	0.313
	TC or TT, n (%)	50 (39.4)	26 (44.1)	24 (35.3)	
	HaeIII-gene				
	CC (wild type), n (%)	78 (61.4)	37 (62.7)	41 (60.3)	0.780
	TC or TT, n (%)	49 (58.6)	22 (37.3)	27 (39.7)	
	Fibrinogen in mg/dl, mean (sd)	280.1 (78.9)	277.5 (72.7)	282 (84.5)	0.729
Hearing recovery	None, n (%)	43 (33.9)	18 (30.5)	25 (36.8)	
	1-10 dB, n (%)	46 (36.2)	19 (32.2)	27 (39.7)	0.160^§^
	> 10 dB, n (%)	38 (29.9)	22 (37.3)	16 (23.5)	

&  =  data missing on 3 patients; &&  =  data missing on 6 patients; CC  =  wild type, TC  =  heterozygous; TT  =  homozygous; §  =  Mantel-Haenszel Chi-square test.

### Blood Samples and Routine Measurements

Blood was drawn by venipuncture at the time when hearing recovery was determined, which was at least 8 weeks after ISSHL to avoid acute phase reactions secondary to the acute event. Fibrinogen was measured in citrated plasma after centrifugation for 15 min at 2,500 g. The sample analysis was done within 1 h after venipuncture. DNA was extracted from EDTA-treated blood by a spin column procedure (Quiagen, Germany).

Fibrinogen was determined using the Multifibren U reagent (BCS analyzer; Dade Behring). The coagulation process was initiated by adding a large excess of thrombin to the plasma sample. The time until formation of a fibrin clot was measured. The fibrinogen concentration was then calculated from a standard curve prepared by measuring fibrinogen calibrators of a known concentration. All other routine measurements were performed as previously described [19].

### Flow Cytometry Analysis

The platelet-surface GPIa receptor density (integrin α_2_β_1_) was determined by flow cytometry analysis using the antibody originally sold by American Diagnostica, which can now be obtained from Biocytex (Marseille, France). Measurements were performed as described by the company (manual reference number 7008). In order to maintain platelet integrity, utmost care was exercised to avoid platelet activation during collection. The anticoagulant used was trisodium citrate 0.129 M (using a ratio 9∶1 volumes). Blood samples were treated within 4 hours after collection. Blood was stored at room temperature before testing (18–25°C). The test was performed on platelet rich plasma (PRP). 25 µL of PRP was diluted and then added to 20 µL of anti GpI, GpIb or GpIIIa mouse antibody. Tubes were gently vortexed for 1 to 2 seconds, secondary polyclonal anti-mouse IgG-FITC antibody was added and incubated at room temperature for 10 minutes, and finally diluted with 2 ml of dilution solution (obtained from the test kit) before measurements were performed within 2 hours of obtaining the samples. Before cytometric analysis on a FACS-STAR (Coulter) cytometer the PRP antibody mixture was stored at 2–8°C. For calibration analysis, beads coated with increasing and accurately known quantities of mouse IgG were stained in parallel with the sample using the same secondary IgG-FITC antibody and incubated for the same amount of time as per the manufacturer's recommendations. The bead population was gated using a discriminator on forward scatter to minimize background. Mean fluorescence intensity values were interpolated on the calibration curve and the number of molecules read off directly. The GpIa, GpIb and GpIIIa platelet-surface receptor densities were determined in parallel in the same experimental setting. According to the manufacturer, the values for negative isotypic controls were very low (∼300 sites per platelet), so that these controls were not included in the test kit. The number of glycoprotein molecules per platelet, as described by the manufacturer for a normal population, was: 53,000±12,000 for GpIIIa, 38,000±11,000 for GpIb, and 5,000±2,800 for GpIa. In the experiments described here, the number of glycoprotein molecules per platelet was in the same range, except for the case of GPIa, in which 6 participants receptor density markedly exceeded this range (>7,000 molecules per platelet). Therefore, these 6 outliers were removed and data on glycoprotein density analysed for the 53 remaining patients.

### Genetic Analysis

#### Fibrinogen polymorphisms

The fibrinogen polymorphisms BcII, Aα312, HaeIII and Dusart that may influence fibrinogen concentration or function [21] were determined by real time PCR, using assay-by-design kits on the TaqMan-system (Applied Biosystems, Weiterstadt, Germany). Sequence information for primers and minor grove binder probes are as follows:

fibrinogen *BcII:*             Sequence

Forward primer             -ATCCAGCAATCTCACTTTTAGGCATAT-

Reverse primer             -GGGCATCATATAAAAGGACCTGAACA-

Probe 1 (labeled with the fluorescent dye VIC)   -CATCTGTGGTCATTGC-

Probe 2 (labeled with the fluorescent dye FAM)   -CATCTGTGATCATTGC-

fibrinogen *Aα312:*

Forward primer             -GAGGGACTGCAACCTGGAAAC-

Reverse primer             -CAGTTCCAGAGCTCCCAGAGTT-

Probe 1 (labeled with the fluorescent dye VIC)   -TGGAAGTACTGGAAGC-

Probe 2 (labeled with the fluorescent dye FAM)   -TGGAAGTGCTGGAAG-

fibrinogen *HaeIII:*

Forward primer             -GAGGGACTGCAACCTGGAAAC-

Reverse primer             -CAGTTCCAGAGCTCCCAGAGTT-

Probe 1 (labeled with the fluorescent dye VIC)   -CATCTGTGGTCATTGC-

Probe 2 (labeled with the fluorescent dye FAM)   -TGGAAGTGCTGGAAG-

fibrinogen *Dusart:*

Forward primer             -CATCACCCTGGGATAGCTGAAT-

Reverse primer             -TCGTGCTACTAGTAAATTGTTTGCTGTA-

Probe 1 (labeled with the fluorescent dye VIC)   -ACCACGGGAAGGG-

Probe 2 (labeled with the fluorescent dye FAM)   -ACCACAGGAAGGGA-

Platelet glycoprotein Ia C807T

Determination of the C or T allele at position 807 of the GPIa gene was performed by mutagenically separated PCR. We determined the C807T genotype indirectly by identifying the linked G873A genotype. The three following primers were used:

Primer 873A in 5` of exon:

5`-TATTCAGCAGAGTCTGGTGGGCGACGAAGTGCTTCA-3`;

Primer 873G in 3` of exon:

5`-CTTCTGGTGGGCGACGAAGTGCTATG-3`;

Primer 1 in 5` of exon:

5`-CTCAGTATATTGTCATGGTTGCATTG-3`;

The DNA fragments were visualized on a 3 or 5% agarose gel after staining with ethidium bromide by ultraviolet transillumination.

### Ethics

The study protocol was reviewed and approved by the ethics committee of the University of Münster, Germany. Written informed consent was obtained from all patients. If participants were minors, written informed consent was obtained from next of kin, caretakers, or guardians.

### Statistical Analysis

Descriptive statistics encompassed the mean (standard deviation) for normally distributed metric variables and the median (range) for not-normally distributed metric variables, respectively. To test for differences between 2 independent subgroups, we used student's t-test for normally distributed metric variables and Mann-Whitney-U-Test for not normally distributed, metric variables. To test for differences between >2 independent ordered groups (of hearing recovery), we used the Mantel-Haenszel Chi-square test in binary and ordinal variables, and ANOVA for normally distributed metric variables respectively Kruskal-Wallis-Test for not-normally distributed metric variables. Binary logistic regression was used to examine the independent association of fibrinogen plasma level, glycoprotein receptor densities and their interaction with hearing recovery >10 dB (averaged over 4 frequencies, 250 Hz, 1,000 Hz, 2,000 HZ, and 4,000 Hz) versus hearing recovery ≤10 dB. We assumed a positive effect of low levels of both fibrinogen and glycoprotein receptors on hearing recovery [22], [23]. Therefore, glycoprotein receptor densities and fibrinogen levels were dichotomized using their mean values, and levels above mean were taken as reference categories. Following the recommendations by Knol and VanderWeele [24], we present odds ratios and their 95% confidence intervals with a single reference category (patients with high fibrinogen levels and high glycoprotein receptor levels) for the association of fibrinogen and glycoprotein receptor density with good hearing recovery. Results of glycoprotein receptor densities were displayed stratified for both levels of fibrinogen. Interaction measures were calculated both on additive (relative excess risk due to interaction, RERI) and on multiplicative scale [24]. As the analyses were explorative, no adjustment for multiple testing was done and the statistical significance was accepted at a level of *p*<0.05.

## Results

### Univariate analysis of platelet integrin α_2_β_1_ (GpIa), GpIb and GpIIIa expression in ISSHL patients and controls

The C807T polymorphism in GpIa is a silent polymorphism: it does not alter the deduced amino acid sequence of the translated protein. However, an association between the expression levels of integrin α_2_β_1_ on platelets and the C807T polymorphism has been suggested [25], raising the possibility that integrin α_2_β_1_ expression may influence ISSHL incidence and/or recovery from ISSHL.

**Figure 1:**
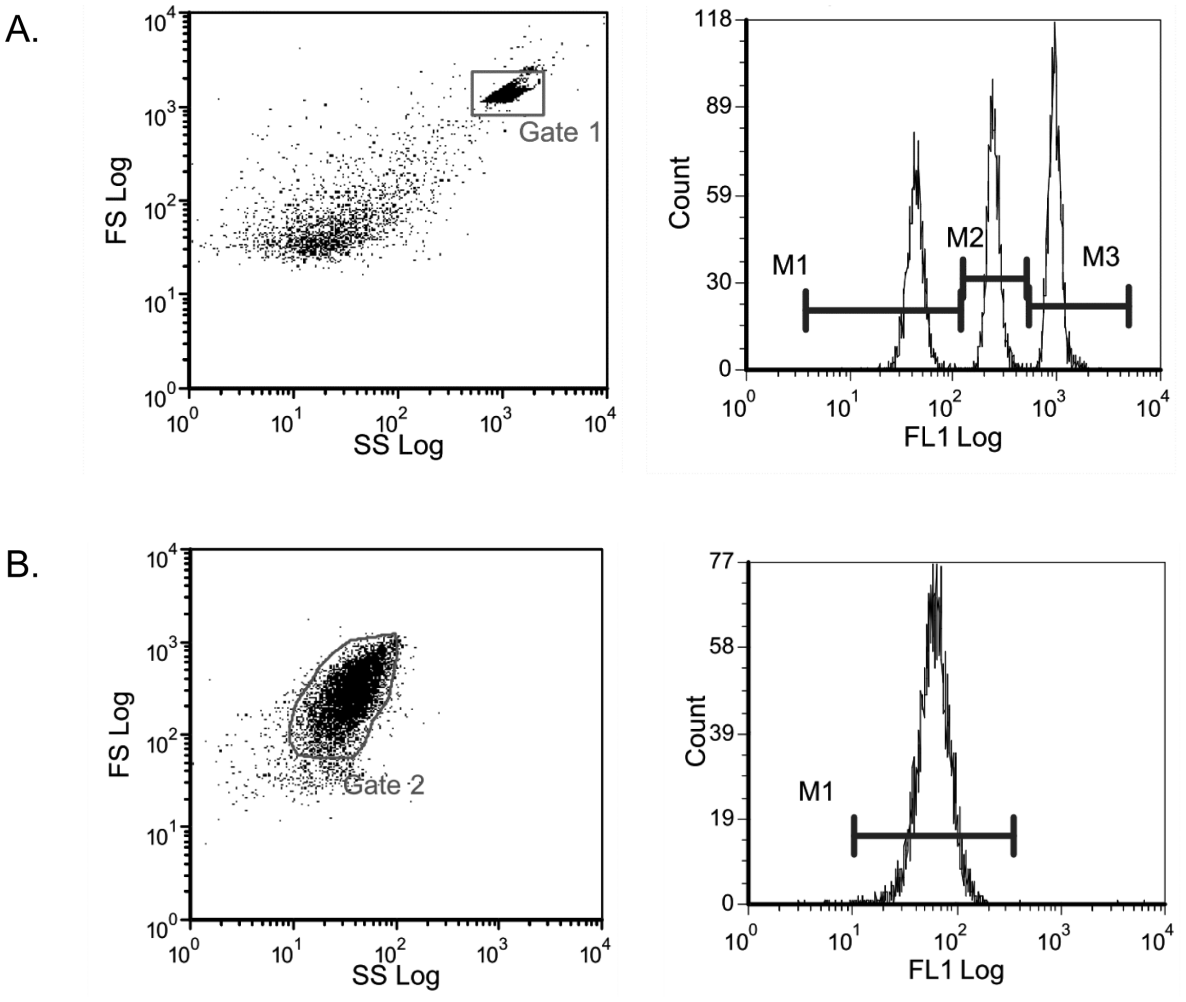
Test calibration cytogram with cursor settings in gated fluorescence histogram (a) and platelet rich plasma cytogram with GpIa immune-labelling cursor settings (b). A single color flow cytometric analysis of the platelet glycoproteins GpIa, GpIb and GpIIIa was used (Biocytex, Marseille, France). The number of antigenic sites is determined by converting the fluorescence intensity into the corresponding number of sites per platelet based on calibrated bead standards (a). In all experiments, more than 90% of the platelets of the PRP were gated for the experiment, as shown here for GpIa (b).

**Figure 2:**
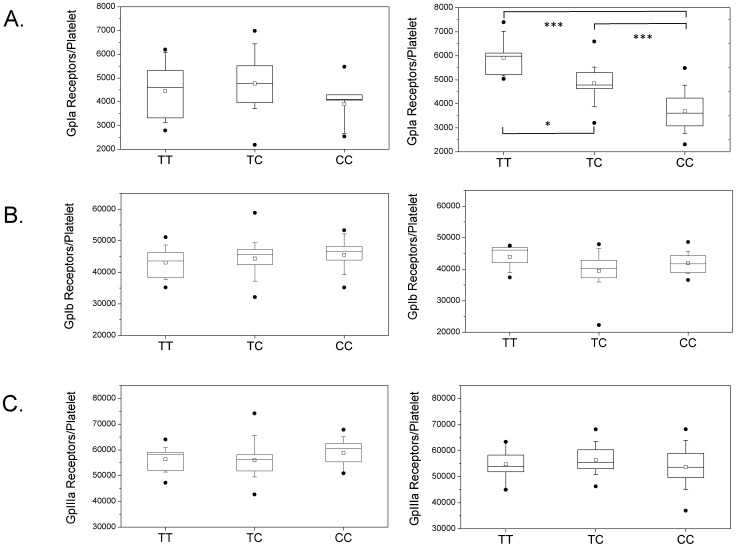
Number of GpIa (a), GpIb (b) and GpIIIa (c) receptor molecules per platelet in patients (left) and controls (right). The values are shown as boxplots, separated for T allele carriers (phenotype TT or CT) and CC allele carriers. The boundary of the box closest to zero indicates the 25^th^ percentile, the line within the box marks the median, the square indicates the mean, and the boundary of the box farthest from zero indicates the 75^th^ percentile. Whiskers above and below the box indicate the 90^th^ and 10^th^ percentiles. The points represent the 1^st^ and 99^th^ percentiles. P indicates the level of significance according to the Mann-Whitney-U-test. *, P<0.05; ***, P<0.001.

The concentration of platelet integrin α_2_β_1_ was measured using a highly sensitive fluorescence-based method (Fig. 1). In healthy age- and sex-matched controls, we could find a significant 1.4-fold higher mean number of integrin α_2_β_1_ molecules per platelet in T-allele carriers compared to non-T-allele carriers (TT carriers 5,916 +/− 888; TC carriers 4,844 +/− 715; CC carriers 3,681 +/− 900; p<0.05 for TT vs. TC; p<0.001 for TT vs. CC and for TC vs. CC; Fig. 2a, right panel). In patients, we found a 1.2-fold higher mean number of integrin α_2_β_1_ molecules per platelet in T-allele carriers compared to non-T-allele carriers (TT carriers 4,446 +/− 1,188; TC carriers 4,766 +/− 1,166; CC carriers 3,905 +/− 1,012; NS for all comparisons; Fig. 2a, left panel). We found no significant difference in the number of integrin α_2_β_1_ molecules per platelet between patients and controls, neither in allele carriers nor in non-allele carriers.

We also measured the platelet expression of the glycoproteins Ib (GpIb), as shown in Fig. 2b, and IIIa (GpIIIa), as shown in Fig. 2c. GpIb, in the form of the platelet glycoprotein Ib/IX/V receptor complex, plays a key role in the platelet binding to von Willebrand Factor and the initial adhesion of platelets to the vessel wall, whereas GpIIIa is, in form of the glycoprotein IIb/IIIa complex, also known as integrin α_IIb_β_3_, an important receptor for fibrinogen [20]. We did not observe an association of GpIa genotype with GpIb or GpIIIa platelet densities. No significant differences in the levels of GpIb or GpIIIa between patients and controls were observed either.

### Univariate analysis of basic patients' characteristics, platelet integrin α_2_β_1_ (GpIa), GpIb, GpIIIa expression, GPIa genotype, fibrinogen concentration, and fibrinogen genotype in ISSHL patients with different hearing recovery

As shown in table 2, after averaging hearing recovery over 4 frequencies (250 Hz, 1,000 Hz, 2,000 Hz, and 4,000 Hz) there was no hearing recovery in 43 (33.9%) study participants while 46 (36.2%) showed a mean hearing recovery up to 10 dB (moderate recovery). Another 38 (29.9%) patients showed hearing recovery of >10 dB (good recovery). The 3 groups did not differ in their basic patients' characteristics except for vertigo: patients with good hearing recovery showed the lowest prevalence of vertigo (24.3%), while in patients with no hearing recovery, vertigo prevalence was 58.5% (p for trend  = 0.002). Fibrinogen plasma levels differed significantly across groups of hearing recovery (p = 0.029), with the highest fibrinogen levels being found in patients with no hearing recovery. However, 3 fibrinogen polymorphisms: the B2 allele of the BclI restriction fragment length polymorphism of the fibrinogen β-chain, the 312Ala (GCT) allele of the Aα312 polymorphism of the fibrinogen α-chain [21] and the β-fibrinogen Hae III (-455 G-A) gene variant did not vary between groups. Similarly, a GpIa C807T polymorphism that may influence concentration and function of GPIa, did not vary between groups of hearing recovery. In a subgroup of 59 patients the expression of platelet GpIa, GpIb and GpIIIa receptor was measured. Significant differences between the 3 groups of hearing recovery were found for GPIIIa expression (p = 0.037).

**Table 2:** Basics characteristics in groups with different hearing recovery, n = 127 respectively n = 59.

Variable		All patients stratified for hearing recovery			
		None	1–10 dB	> 10 dB	p
		n = 43 (33.9%)	n = 46 (36.2%)	n = 38 (29.9%)	
Basic patients' characteristics	Age in years, mean (sd)	53.4 (16.8)	52.8 (19.1)	53.8 (15.3)	0.966^#^
	Female gender, n (%)	25 (58.1)	21 (45.7)	16 (42.1)	0.146^§^
	Smoker, n (%)^&^	21 (48.8)	24 (54.6)	23 (62.2)	0.236^§^
	Tinnitus, n (%)^&&^	30 (75.0)	32 (72.7)	29 (78.4)	0.741^§^
	Vertigo, n (%)^&&^	24 (58.5)	13 (29.6)	9 (24.3)	**0.002^§^**
Laboratory values	GPIa-gene				
	CC (wild type), n (%)	29 (67.4)	31 (67.4)	30 (79.0)	0.268^§^
	TC or TT, n (%)	14 (32.6)	15 (23.6)	8 (21.1)	
	BcLI-gene				
	CC (wild type), n (%)	27 (62.8)	29 (63.0)	26 (68.4)	0.606^§^
	TC or TT, n (%)	16 (37.2)	17 (37.0)	12 (31.6)	
	A312-gene				
	CC (wild type), n (%)	28 (65.1)	27 (58.7)	22 (57.9)	0.501^§^
	TC or TT, n (%)	15 (34.9)	19 (41.3)	16 (42.1)	
	HaeIII-gene				
	CC (wild type), n (%)	26 (60.5)	26 (56.5)	26 (68.4)	0.485^§^
	TC or TT, n (%)	17 (39.5)	20 (43.5)	12 (31.6)	
	Fibrinogen in mg/dl, mean (sd)	306.0 (91.5)	266.6 (62.2)	267.2 (76.3)	**0.029^$^**
Receptor density	GPIa, mean (SD), n = 53^$$^	4,162.9 (1,398.1)	4,929.9 (1,104.1)	4,590.6 (940.2)	0.169**^$^**
(n = 59/53)	GPIb, mean (SD)	43,604.1 (5,099.6)	45,947.0 (5,361.6)	43,300.1 (5,118.5)	0.239**^$^**
	GPIIIa, mean (SD)	56,658.1 (5,433.4)	58,976.9 (6,377.1)	54,190.3 (5,516.4)	**0.037^$^**

Sd  =  standard deviation; #  =  Kruskal-Walis-Test; &  =  data missing on 3 patients; &&  =  data missing on 6 patients; §  =  Mantel-Haenszel Chi-square test; CC  =  wild type, TC  =  heterozygous; TT  =  homozygous; $  =  ANOVA, $$  =  6 outlier removed.

### Modification of the effect of platelet integrin α_2_β_1_ (GpIa), GpIb, GpIIIa expression on hearing recovery by fibrinogen in ISSHL patients

As shown in tables 3-5, after adjustment for smoking and vertigo, lower levels of all three platelet glycoproteins (except for GpIa in patients with fibrinogen below mean) were associated with better hearing recovery although this association was significant solely for GPIb in patients with fibrinogen levels above the mean (table 4). As shown in table 3, fibrinogen modified the effect of GPIa receptor density on hearing recovery: in participants with fibrinogen levels below the mean, lower GPIa receptor levels were associated with decreased probability of hearing improvement, while in patients with fibrinogen levels above the mean, the probability of hearing recovery was increased. The modification effect of fibrinogen on the association of GPIa receptor level and hearing recovery was significant on a multiplicative scale (ratio of ORs. 0.47 (95% confidence interval: 0.22 – 0.99; p = 0.048). For GPIb, no such effect modification by fibrinogen was observed (table 4): in both strata of fibrinogen levels, the effect of GPIb receptor density on hearing recovery was nearly identical (OR = 2.00 versus OR = 1.98, ratio of ORs = 0.99 (95% confidence interval: 0.52 – 1.86; p = 0.968). Fibrinogen did not modify the effect of GPIIIa on hearing recovery either (table 5): the ratio of ORs was 1.16 (95% confidence interval: 0.61 – 2.20; p = 0.650). In all three platelet glycoproteins, there was also no indication of a significant positive effect modification of platelet glycoprotein receptor density across strata of fibrinogen on an additive scale.

**Table 3:** Results of binary logistic regression analyses, dependent variable  =  hearing improvement >10 dB, independent variables  =  GPIa receptor density and fibrinogen, n = 49^#^.

	GPIa receptor density above mean		GPIa receptor density below mean		ORs (95%-CI) for GPIa receptor density within different strata of fibrinogen
	N with/without hearing improvement	OR (95% CI)	N with/without hearing improvement	OR (95% CI)	
Fibrinogen above mean	1/9	1.0	5/10	1.24 (0.59 – 2.60); p = 0.568	1.24 (0.59 – 2.60); p = 0.568
Fibrinogen below mean	8/13	1.50 (0.71 – 3.14); p = 0.286	5/17	0.84 (0.29 – 2.42); p = 0.741	0.56 (0.25 – 1.24); p = 0.150

Measure of effect modification on additive scale: relative excess risk due to interaction, RERI (95% CI)  =  −0.90 (−1.97 – 0.17)

Measure of effect modification on multiplicative scale: ratio of ORs (95% CI) = 0.45 (0.21 – 0.94), p = **0.034**

ORs are adjusted for smoking and vertigo, #  =  4 datasets missing due to missing covariates.

**Table 4:** Results of binary logistic regression analyses, dependent variable  =  hearing improvement > 10 dB, independent variables  =  GPIb receptor density and fibrinogen, n = 55^#^.

	GPIb receptor density above mean		GPIb receptor density below mean		ORs (95%-CI) for GPIb receptor density within different strata of fibrinogen
	N with/without hearing improvement	OR (95% CI)	N with/without hearing improvement	OR (95% CI)	
Fibrinogen above mean	3/14	1.0	4/9	2.00 (1.04 – 3.86); p = **0.038**	2.00 (1.04 – 3.86); p = **0.038**
Fibrinogen below mean	4/15	1.28 (0.68 – 2.41); p = 0.453	10/17	2.52 (0.96 – 6.64); p = 0.061	1.98 (0.88–4.43); p = 0.098

Measure of effect modification on additive scale: relative excess risk due to interaction, RERI (95% CI)  =  0.24 (−1.46 – 1.95)

Measure of effect modification on multiplicative scale: ratio of ORs (95% CI) = 0.99 (0.52– 1.86), p = 0.968

ORs are adjusted for smoking and vertigo, #  =  4 datasets missing due to missing covariates.

**Table 5:** Results of binary logistic regression analyses, dependent variable  =  hearing improvement > 10 dB, independent variables  =  GPIIIa receptor density and fibrinogen, n = 55^#^.

	GPIIIa receptor density above mean		GPIIIa receptor density below mean		ORs (95%-CI) for GPIIIa receptor density within different strata of fibrinogen
	N with/without hearing improvement	OR (95% CI)	N with/without hearing improvement	OR (95% CI)	
Fibrinogen above mean	2/11	1.0	5/12	1.57 (0.81 – 3.03); p = 0.178	1.57 (0.81 – 3.03); p = 0.178
Fibrinogen below mean	5/17	1.44 (0.76 – 2.70); p = 0.260	9/15	2.62 (0.96 – 7.15); p = 0.060	1.82 (0.83 – 4.01); p = 0.135

Measure of effect modification on additive scale: relative excess risk due to interaction, RERI (95% CI)  =  0.61 (−1.11 – 2.34)

Measure of effect modification on multiplicative scale: ratio of ORs (95% CI) = 1.16 (0.61– 2.20), p = 0.650

ORs are adjusted for smoking and vertigo, # = 4 datasets missing due to missing covariates.

## Discussion

The C807T polymorphism has previously been shown to be a risk factor for incidence of ISSHL [19] and other acute thrombotic events like myocardial infarction, stroke and retinal vein thrombosis, particularly in younger patients [30], [31]. In this study, the polymorphism C807T directly correlated with a higher number of α_2_β_1_ molecules on the platelet surface of patients and controls, suggesting a direct amplifying influence of the C807T polymorphism on α_2_β_1_ integrin expression. These data are in accordance with a previous study [25] and support the model that the ‘silent’ mutation C807T in GpIa significantly influences platelet expression of α_2_β_1_ integrin. However, this effect was moderate (1.2-1.4-fold) and significant only in controls. Moreover, we found no significant difference in the number of integrin α_2_β_1_ molecules per platelet between patients and controls, neither in allele carriers nor in non-allele carriers. We had also no indication for any secondary effects of GpIa genotype on concentrations of other platelet glycoproteins, or for aberrant levels of GpIb or GpIIIa in ISSHL patients_._ These findings suggest that, certainly beyond the critical acute phase, receptor densities are not different between ISSHL patients and healthy controls. A higher GpIa expression in T allele carriers is *per se* apparently not sufficient to trigger an ISSHL.

Fibrinogen level (p = 0.029), vertigo (p = 0.002) and lower GpIIIa receptor density (p = 0.037) varied between groups with different hearing recovery.

We found a significant association between lower plasma fibrinogen levels and good hearing recovery. However, there was no significant association of fibrinogen polymorphisms (that may influence fibrinogen concentration or function) with hearing recovery, suggesting that this effect might have resulted from factors other than genetics. Suckfüll et al showed that fibrinogen apheresis improves the outcome in ISSHL patients [16]. This observation and the current study suggest that higher fibrinogen is not only a surrogate marker of increased ISSHL risk, but a pathogenetic factor in the development of ISSHL itself.

Fibrinogen can be influenced by various non-genetic factors, which include: smoking, obesity, diabetes, hormones, and acute inflammation or trauma [27], [28]. It was remarkable that the majority of ISSHL patients in this investigation were smokers. Smoking may have influenced fibrinogen plasma concentration, platelet function [28], [29] and hearing recovery. However, in univariate analysis, the percentage of smokers was rather (non significantly) higher in groups with better hearing recovery. We have no explanation for this apparent paradox.

Hearing impairment in ISSHL is often accompanied by vertigo [4], which is caused by a disturbed vestibular system. The blood supply to the inner ear originates from the vertebrobasilar system, and vertebrobasilar ischemic stroke can present with vertigo and hearing loss due to infarction of the inner ear [26]. Thus, one possible explanation for vertigo experienced by ISSHL patients with poor prognosis (no or only limited recovery) might be an expression of a more extended degree of thrombotic damage.

The possible positive effect of low platelet concentrations of GPIIIa on hearing recovery in ISSHL patients has been shown for the first time in this study. The Glycoprotein IIb/IIIa complex (also known as integrin α_IIb_β_3_) is a receptor for fibrinogen and aids in platelet activation. It is a target of several well-established drugs including abciximab, eptifibatide and tirofiban, which are used in treating patients who have unstable angina, certain types of heart attacks, and are used in combination with angioplasty with or without stent placement. Further studies are recommended to prove possible beneficial effects of these drugs in ISSHL. Additionally, because ADP induces exposure of fibrinogen-binding determinants on GpIIb/IIIa complexes, ADP antagonists such as clopidogrel, ticlopidine or prasugrel may have positive effects in ISSHL.

After adjustment for smoking and vertigo, lower levels of all three platelet glycoproteins were associated with better hearing recovery, although this association was significant solely for GPIb.

GpIb, as component of the GpIb/V/IX complex, binds von Willebrand factor and allows platelet adhesion and plug formation at sites of vascular injury. However, there have been conflicting reports on a possible association of polymorphisms of this glycoprotein with myocardial infarction and coronary artery disease [32]–[39]. A possible positive effect of low platelet concentrations of GPIb on hearing recovery has not been described before.

Fibrinogen significantly modified the effect of GPIa receptor density on hearing recovery, but in an unexpected manner: a putative protective effect of low GpIa concentrations, after adjustment for vertigo and smoking, was significantly diminished in patients with lower fibrinogen levels. There was a harmful effect modification by fibrinogen on a multiplicative scale. The origin of this effect is unclear. Apart from complex interactions between fibrinogen and GpIa [22], it is possible that pathogenetic factors other than fibrinogen may play a major role in these cases and that protective effects of lower GpIa levels become less significant when plasma fibrinogen is not elevated. A possible interrelationship between GpIa and fibrinogen is of interest insofar as GpIa, as GpIa/IIa complex, functions as receptor engaged in platelet adhesion to collagen and is believed to be associated with the risk of myocardial infarction or ischemic stroke [30], [31]. In contrast to GpIa, the protective effect of lower GPIb and GPIIIa receptor density on hearing recovery was not modified by fibrinogen.

Limitations of our study are the relatively small number of patients for whom glycoprotein receptors were measured and the relative high percentage of smokers, both of which may have influenced and biased results. The power of this study was not sufficient to allow for sensitivity analyses in subgroups (e.g. in smokers/non-smokers) or for more complex analyses of interactions between genetics, environmental factors and platelet glycoproteins. Another meaningful supplement to our analysis would have been evaluation of hearing recovery in speech audiometry. Other investigators were able to show that speech audiometry might be more sensitive in detecting differences in hearing recovery [16].

Altogether, data from this study further suggest a vascular/rheological origin of ISSHL with unique features of thrombosis in the inner ear artery that may include a complex interrelationship among platelet glycoproteins and plasma fibrinogen. Further studies with prospective design are necessary to confirm and deepen the findings described here and to prove the proposed therapeutic options, particularly glycoprotein blockade.
